# The association between statin therapy during intensive care unit stay and the incidence of venous thromboembolism: a propensity score-adjusted analysis

**DOI:** 10.1186/2050-6511-14-57

**Published:** 2013-11-11

**Authors:** Shmeylan A Al Harbi, Mohammad Khedr, Hasan M Al-Dorzi, Haytham M Tlayjeh, Asgar H Rishu, Yaseen M Arabi

**Affiliations:** 1 College of Pharmacy, King Saud bin Abdulaziz University for Health Sciences, King Abdulaziz Medical City, Riyadh, Saudi Arabia; 2 College of Medicine, King Saud bin Abdulaziz University for Health Sciences, King Abdulaziz Medical City, MC 1425, PO Box 22490, Riyadh 1426, Saudi Arabia; 3 King Abdulaziz Medical City, Riyadh, Saudi Arabia

**Keywords:** Venous thromboembolism, Outcome assessment, Intensive care, Hospital mortality, Propensity scores, Statins

## Abstract

**Background:**

Studies have shown that statins have pleiotropic effects on inflammation and coagulation; which may affect the risk of developing venous thromboembolism (VTE). The objective of this study was to evaluate the association between statin therapy during intensive care unit (ICU) stay and the incidence of VTE in critically ill patients.

**Methods:**

This was a post-hoc analysis of a prospective observational cohort study of patients admitted to the intensive care unit between July 2006 and January 2008 at a tertiary care medical center. The primary endpoint was the incidence of VTE during ICU stay up to 30 days. Secondary endpoint was overall 30-day hospital mortality. Propensity score was used to adjust for clinically and statistically relevant variables.

**Results:**

Of the 798 patients included in the original study, 123 patients (15.4%) received statins during their ICU stay. Survival analysis for VTE risk showed that statin therapy was not associated with a reduction of VTE incidence (crude hazard ratio (HR) 0.66, 95% confidence interval (CI) 0.28-1.54, P = 0.33 and adjusted HR 0.63, 95% CI 0.25-1.57, P = 0.33). Furthermore, survival analysis for hospital mortality showed that statin therapy was not associated with a reduction in hospital mortality (crude HR 1.26, 95% CI 0.95-1.68, P = 0.10 and adjusted HR 0.98, 95% CI 0.72-1.36, P = 0.94).

**Conclusion:**

Our study showed no statistically significant association between statin therapy and VTE risk in critically ill patients. This question needs to be further studied in randomized control trials.

## Background

Venous thromboembolism (VTE), encompassing deep vein thrombosis (DVT) and pulmonary embolism (PE), is a common complication of critical illness and is associated with significant morbidity and mortality [1,2]. In general, the incidence of VTE is 1–2 per 1,000 individuals per year [3] and reaches 1% per year in those aged over 70 years [4]. In critically ill patients, the incidence has been reported up to 10% despite thromboprophylaxis [2]. Statins (Hydroxy-3- methylglutaryl conenzyme A reductase inhibitors) have demonstrated efficacy in reducing cholesterol levels and improving cardiovascular outcomes when administered for both primary and secondary indications. Additionally, through their effects on inflammation and coagulation [5], statins may have antithrombotic properties that can affect not only arterial but also venous thrombosis. Although, venous and arterial thrombosis have largely been considered separate diseases [6-8], multiple studies have suggested that they both share certain risk factors, pathogenesis and possibly statin effects [9-11]. The antithrombotic properties of statins may be related to decreasing platelet aggregation, inhibition of tissue factor and plasminogen activator-inhibitor 1 expression, increasing expression of tissue plasminogen activator activity, and increasing expression of thrombomodulin that activates protein C and prevent thrombin-induced platelet and factor V activation and fibrinogen clotting [12,13]. Clinical studies have yielded variable estimates of the statin effect on VTE [14-19]. Observational studies in outpatient population as well as one randomized controlled trial in healthy older adults have suggested a protective effect of statin therapy [14-17]. In critically ill medical-surgical patients, there are limited studies examining this association. Therefore, we sought to assess the association between statins and VTE incidence in a cohort of critically ill patients.

## Methods

### Setting

The study was conducted in the adult medical-surgical intensive care unit (ICU) of King Abdulaziz Medical City, a tertiary care academic referral center in Riyadh, Saudi Arabia. The ICU admits medical, surgical, and trauma patients, and operates as a closed unit with 24-hr, 7-day onsite coverage by critical care board certified intensivists [20]. The nurse-to-patient ratio in the unit is approximately 1:1.2.

### Study design

This is a post-hoc analysis of a recently published cohort study of the effect of mechanical thromboprophylaxis, intermittent pneumatic compression (IPC) or graduated compression stocking (GCS) on the incidence of VTE in patients admitted to the ICU between July 2006 and January 2008 [21]. The original study included 798 patients. Inclusion criteria were age ≥18 years and expected ICU length of stay of more than 48 hours. Patients were excluded if they were on therapeutic anticoagulation with warfarin or heparin, admitted to the ICU with acute PE, DVT, or had do-not-resuscitate or brain death status on or within first 24 hours of ICU admission. The patients were followed for a total of 30 days from admission to ICU. The study was approved by institutional review board of the hospital. The analysis included all patients who were in the original cohort study. Informed consent was not required.

### Statin therapy

Data about statin therapy in the ICU were collected from the ICU pharmacy database and were matched and combined to the original clinical study database [21]. Statins were continued if they had been prescribed in the pre-ICU period or could have been initiated in the ICU for patients admitted with stroke or acute coronary syndrome. Dosage was at the discretion of the treating physician.

### Data collection

We used the following data for the analysis: age, Acute Physiology and Chronic Health Evaluation (APACHE II) score [22], admission Glasgow coma scale (GCS) score, creatinine, international normalized ratio (INR), activated partial thromboplastin time (aPTT), diagnosis of trauma, femur fracture, presence of central line, bedridden status for more than 3 days whether this was at home or in the hospital, malignancy, recent surgery, previous VTE, presence of hemodialysis catheter, the use of graduated compression stocking, the use of intermittent pneumatic compression device, the use of unfractionated heparin or enoxaparin, packed red blood cells (PRBC) and platelet transfusion. The use of aspirin was tested later in a separate analysis.

### Outcomes

The primary outcome was the effect of statin therapy on VTE incidence (lower extremities DVT, PE, or both) during the ICU stay and up to 5 days after ICU discharge. Overall 30-day hospital mortality was secondary outcome. In this study, VTE was clinically ascertained and confirmed either by Doppler ultrasound for DVT or by helical chest tomography for PE.

### Statistical analysis

Due to the non-random allocation of study groups, propensity scores were used to balance baseline characteristics (Table 1). The scores were derived from a logistic regression model using “pscore” program in Stata/SE version 11 for Windows (StatCorp LP, College Station, TX, USA). The model satisfied Hosmer-Lemeshow goodness-of-fit test (P = 0.41) and showed excellent discrimination ability (the area under ROC = 84%). The derived propensity scores were then divided into 6 blocks and used later in analysis as stratification factor or as an adjusting covariate. Variables included in the propensity score generation model were selected according to their relationship to the outcome (VTE) rather than the exposure (statin therapy). However, some of these variables were also related to the exposure. This approach is one of three possible ways (related to exposure and outcome, related to outcome alone, and related to exposure alone) for variable selection. It has been shown to reduce bias and variance of estimated exposure effect [23,24]*.* Those variables were: age, APACHE II score, GCS, diagnosis of trauma, presence of femur fracture, creatinine level, INR, aPTT level, central venous line presence, history of malignancy, recent surgery, history of previous VTE, PRBC and platelet transfusion, hemodialysis catheter use, use of graduated compression stocking, use of intermittent pneumatic compression device, and unfractionated heparin or enoxaparin.

**Table 1:** Baseline characteristics of the statins and non-statin therapy groups

	**Statin (n =123)**	**Non-Statin (n = 675)**	***P*-value**	***PS Adjusted P-Value***
Age, mean ± SD, years	67.1 ± 11.3	47.1 ± 21.1	<0.001	0.59
APACHE II, mean ± SD	26.7 ± 8.1	23.5 ± 9.1	0.0002	0.90
GCS, mean ± SD,	9.0 ± 4.6	8.5 ± 4.0	0.20	0.96
Creatinine, mean ± SD, μmol/L^*^	228.0 ± 179.3	146.4 ± 133.8	<0.001	0.81
INR, mean ± SD	1.3 ± 0.5	1.4 ± 0.7	0.03	0.93
aPTT, mean ± SD,	43.4 ± 57.1	42.2 ± 60.8	0.83	1.00
Trauma, No%	3 (2.4)	223 (33.0)	<0.001	0.007
Femur fracture, No.%	2 (1.6)	50 (7.4)	0.02	0.45
Any central line present, No. (%)	91(74.0)	504 (74.7)	0.87	0.78
Bedridden for > 3 days, No. (%)	84 (68.3)	310 (45.9)	<0.001	0.44
Malignancy, No. (%)	8 (6.5)	86 (12.7)	0.05	0.77
Recent surgery, No. (%)	22 (17.9)	221(32.7)	0.001	0.65
Previous VTE, No. (%)	4 (3.3)	8 (1.2)	0.08	0.98
Hemodialysis catheter, No. (%)	33 (26.8)	125 (18.5)	0.03	0.71
Compression stocking, No. (%)	26 (21.1)	172 (25.5)	0.31	0.79
Sequential compression device, No. (%)	29 (23.6)	227 (33.6)	0.03	0.75
Unfractionated heparin, No. (%)	97 (78.9)	405 (60.0)	<0.001	0.58
Enoxaparin, No. (%)	16 (13.0)	212 (31.4)	<0.001	0.21
Platelet transfusion, No. (%)	12 (9.8)	132 (19.6)	0.009	0.97

P-values are provided for the differences between the two groups significant before and after propensity score adjustment.

*APACHE*: Acute physiology and chronic health evaluation, *GCS*: Glasgow coma scale, *INR*: International normalized ratio.

*aPTT*: activated partial thromboplastin time, *VTE*: Venous thromboembolism, *PS*: propensity score.

*To convert to conventional units in mg/dL, divide by 88.4.

For hospital mortality analysis, follow-up time was censored at 30 days or at the time of hospital discharge if less than 30 days. Cox-proportional hazard regression was used to evaluate the effect of statins on the incidence of VTE. In addition to crude model, propensity score stratified, propensity score-adjusted and multivariate-adjusted models were assembled for verification. The potential cofounder effect of aspirin use was tested with multivariate models for both VTE and hospital mortality. Hazard ratios (HR) were derived and presented with their 95% confidence intervals (CI). All tests were considered significant at 0.05 alpha level.

## Results

### Patient’s characteristics

Baseline characteristics are shown in Table 1. Of the 798 patients enrolled in the study, 123 (15.4%) received statins during their ICU stay and 57 (7.1%) patients developed VTE (Table 2). Patients who received statins were more likely to be bedridden and had higher BMI. In contrast, non-statin therapy group were more likely to be admitted with the diagnosis of trauma. Atorvastatin was used in 100 patients (81%) at doses 10 to 40 mg/day and simvastatin was used in 23 patients (19%) at 20 mg/day.

**Table 2:** Distribution of hospital mortality and VTE cumulative incidence according to statin use

Statin use*	Hospital mortality**	Incident VTE**
**(n,%)**	**n (%)**	**n (%)**
Yes (123, 15.4%)	58(47.2%)	6 (7.6%)
No (675, 84.6%)	256 (38%)	51 (4.9%)
Total (798)	314 (39.4%)	57 (7.1%)

*Numbers between parentheses reflect counts and percentages, respectively.

**Numbers between parentheses reflect percentage within statin category.

VTE occurred in 6 (7.6%) patients in the statin therapy group and 51 (4.9%) patients in the non-statin therapy group (Table 2). The median follow-up time for statin-therapy and non statin-therapy groups were 17 days (IQR 7–30) and 14 days (IQR 7–26), respectively.

**Figure 1:**
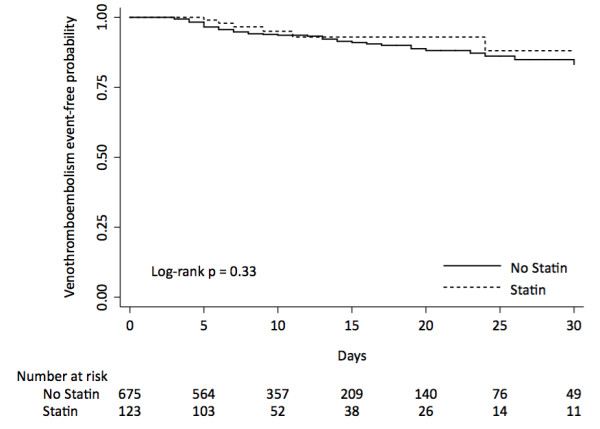
Kaplan-Meier curve and at risk table of the effect of statin use on venothromboembolism incidence in patients admitted to ICU.

Statins were not associated with reduced VTE incidence on univariate analysis (HR 0.66, 95% CI 0.28-1.54, P = 0.33) and on propensity score stratified analysis (HR 0.63, 95% CI 0.25-1.57, P = 0.33) (Table 3 and Figure 1). The analyses using propensity score as an adjustment variable and multivariate analysis revealed similar findings. Adding aspirin as covariate to the multivariate model did not alter the results.

**Table 3:** Crude and PS stratified analysis of VTE risk and Hospital Mortality in statin and non-statin groups

**Type of analysis**	**HR**	**SE**	**95% CI**	**P-value**
**VTE risk**				
Crude analysis	0.66	0.29	(0.28-1.54)	0.33
PS stratified analysis*	0.63	0.29	(0.25-1.57)	0.33
**Hospital Mortality**				
Crude analysis	1.26	0.18	(0.95-1.68)	0.10
PS stratified analysis*	0.98	0.16	(0.72-1.36)	0.94

*Adjusted for age, APACHE II score, GCS, creatinine, INR, aPTT, Trauma, femoral fracture, central line presence, malignancy, recent surgery, previous VTE, hemodialysis catheter use, use of graduated compression stocking, use of sequential compression device, DVT prophylaxis with unfractionated heparin or enoxaparin, and platelet transfusion.

### Hospital mortality

**Figure 2:**
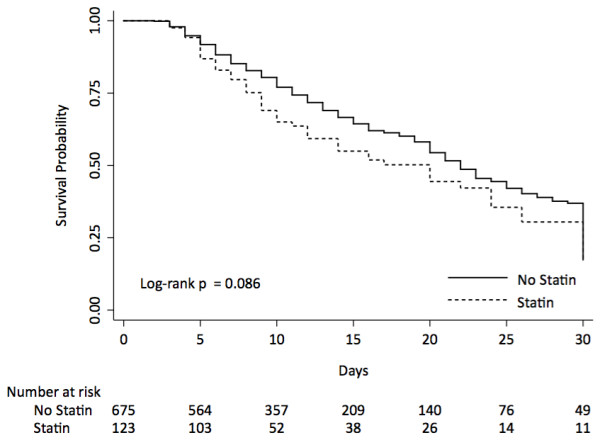
Kaplan-Meier curve and risk table of the effect of statin use on hospital mortality in patients admitted to ICU.

During the study, there were 58 (47.2%) deaths in statin-therapy group and 256 (38%) deaths in no-statin therapy group (Table 2). Statin therapy was not associated with reduction of hospital mortality on crude analysis (HR 1.26, 95% CI 0.95-1.68, P = 0.10) (Table 3 and Figure 2) or on stratified propensity score analysis (HR 0.98, 95% CI 0.72-1.36, P = 0.94). The analyses using propensity score as an adjustment variable and multivariate analysis revealed similar findings. Adding aspirin to the multivariate model did not alter the result.

## Discussion

Our data failed to show a statistically significant reduction in VTE incidence and hospital mortality by continuing statin therapy during patients stay in ICU.

Several other studies have shown that statin therapy reduces VTE incidence but in outpatient settings. The Heart and Estrogens/Progestin Replacement Study of postmenopausal women with heart disease was the first to indicate a relationship between statin therapy and the reduction in VTE risk, showing >50% decrease in VTE events among statin users compared with non-users (HR 0.45, 95% CI 0.23-0.88) [14]. Likewise, Doggen et al. found that statin therapy, among postmenopausal women, was associated with reduction in VTE risk (odds ratio [OR] 0.64, 95% CI 0.39-1.07) [15]. The association was observed in patients on simvastatin (OR 0.51, 95% CI 0.29-0.91) but not in patients on pravastatin (OR1.85, 95% CI 0.65-5.26) [15]. In a retrospective cohort study (N = 125, 862) over 8 years, Ray et al. found that statins were associated with significant DVT risk reduction (HR 0.78, 95% CI 0.69-0.87) among outpatient individuals aged ≥ 65 years [16]. In an age and sex- matched case–control study of hospitalized patients, Lacut et al. found that statin therapy was associated with a significant reduction in the risk of VTE (OR 0.42, 95% CI 0.23-0.76) [25]. In another population-based case–control study, Sorensen et al. found reduction in VTE risk (relative risk of 0.74, 95% CI (0.63-0.85)) in current statin therapy, which was observed whether simvastatin, pravastatin or atorvastatin was used [26]. Further, Ramcharan et al. found, in another population-based case–control study, that the current statin therapy was associated with lower risk of VTE in patients aged 18–70 years (adjusted OR 0.45, 95% CI 0.36-0.56) and this association was observed in all statins, including simvastatin, pravastatin, atorvastatin, fluvastatin, and rosuvastatin [27].

In contrast, two retrospective studies failed to demonstrate a benefit of statin therapy on VTE risk reduction. Yang et al. found no association between current, recent, or past statin therapy and the risk of VTE (OR 1.1, 95% CI 0.3-4.3) [18]. In a large population-based cohort study with a median follow-up period of 4.4 years using a propensity score-based adjustment, Smeeth et al. showed no VTE protective effect with statin therapy (adjusted HR 1.02, 95% CI 0.88-1.18) [19].

Recently, JUPITER trial (Justification for the Use of statins in Prevention: an Intervention Trial Evaluating Rosuvastatin) showed that rosuvastatin (20 mg/day) was associated with lower VTE incidence compared to placebo (HR 0.57, 95% CI, 0.37-0.86) [17].

Three meta-analyses were published on the association of statin therapy in prevention of VTE. Agarwal et al. included one randomized controlled study (JUPITER) and nine observational studies (N = 971patients) and showed that statin therapy was associated with significant reduction in VTE risk (OR 0.68, 95% CI 0.54-0.86), DVT (OR 0.59, 95% CI 0.43-0.82), and PE (OR 0.70, 95% CI 0.53-0.94) [28]. Squizzato et al. included three randomized controlled trials, three cohort and eight case–control studies (N = 836 patients) [29] and demonstrated that statin therapy was associated with lower VTE risk (OR 0.81, 95% CI 0.66-0.99) [29]. Pia et al. included four cohort studies and four case–control studies found that statins were associated with lower risk for VTE (OR 0.67, 95% CI 0.53-0.84) and for DVT (OR 0.53, 95% CI 0.22-1.29) [30].

These studies, which showed benefit of statin therapy in reducing VTE risk, share long-term intervention in a low VTE risk outpatient population, and therefore are not applicable to ICU patients. Our study differs as it evaluates the effect of statin therapy on the short-term occurrence of VTE in a high-risk population.

In contrast to our study, a secondary analysis of the PROTECT (Prophylaxis of Thromboembolism in Critical Care) trial, published as an abstract, found a reduced risk for proximal leg DVT for any statin exposure in the week preceding enrolment (HR 0.46, 95% CI 0.27-0.77) [31]. Our study showed an adjusted HR for the association between statin therapy and VTE of 0.63, (95% CI of 0.25-1.57, P = 0.33). The direction of the point estimate towards protective effect of statins and the magnitude of the association are similar to previous studies in non-critically ill patients. Accordingly, one cannot dismiss beneficial effect of statin therapy on VTE risk during ICU stay, and therefore, further studies are required.

Among the limitations of our study are its monocenter nature, post-hoc design, and the lack of data on the duration of statin therapy prior to ICU admission, and statin side effects. Due the observational nature of the study, the presence of unobserved confounders and competing risks cannot also be entirely excluded. In our pragmatic approach for case ascertainment, we did not include surveillance ultrasounds to detect DVT. Our approach was based on clinical suspension and confirmation with Doppler ultrasound. Therefore, it is likely that some non-clinically evident DVTs were missed. This approach, currently, represents the standard of care and it has been shown to be more cost effective than surveillance approach [32].

We believe that these finding should trigger a larger well-designed randomized-controlled trial in critically ill patients. Such trial should evaluate the dose-effect relationship, the effect of duration of statin therapy on VTE risk, the mechanism of action whether related to the pleiotropic or lipid-lowering effect of statins and whether statins have an additive effect to anticoagulants. We hope that the ongoing clinical trials answer some of these questions [33-35] although trials in critically ill patients are still needed.

## Conclusions

Our study failed to show a statically significant effect of continuing statin therapy during ICU stay on VTE risk and 30-day hospital mortality in critically ill patients. We suggest examining this important question in a randomized, control trial.

## Abbreviations

VTE: Venous thromboembolism; DVT: Deep vein thrombosis; PE: Pulmonary embolism; INR: International normalized ratio; aPPT: Activated partial thromboplastin time; APACHE II: Acute Physiology and Chronic Health Evaluation.

## Competing interests

The authors have no financial or non-financial competing interests to declare.

## Authors’ contribution

SAA: Conception and design, analysis and interpretation of data, drafting of the manuscript, critical revision of the manuscript for important intellectual content, supervision and final approval of the version to be published. MKH: Analysis and interpretation of data, drafting of the manuscript, critical revision of the manuscript for important intellectual content and final approval of the version to be published. HMD: Analysis and interpretation of data, drafting of the manuscript, critical revision of the manuscript for important intellectual content and final approval of the version to be published. HMT: Analysis and interpretation of data, critical revision of the manuscript for important intellectual content and final approval of the version to be published. AHR: Acquisition of data, critical revision of the manuscript for important intellectual content, and final approval of the version to be published. YMA: Conception and design, statistical analysis, critical revision of the manuscript and overall supervision. All authors read and approved the final manuscript.

## Pre-publication history

The pre-publication history for this paper can be accessed here:

http://www.biomedcentral.com/2050-6511/14/57/prepub
